# Microspore culture reveals high fitness of *B*. *napus*-like gametes in an interspecific hybrid between *Brassica napus* and *B*. *oleracea*

**DOI:** 10.1371/journal.pone.0193548

**Published:** 2018-03-01

**Authors:** Qinfei Li, Yangui Chen, Fang Yue, Wei Qian, Hongyuan Song

**Affiliations:** 1 College of Horticulture and Landscape, Southwest University, Chongqing, China; 2 College of Agronomy and Biotechnology, Southwest University, Chongqing, China; Chungnam National University, REPUBLIC OF KOREA

## Abstract

The strategies of crossing *B*. *napus* with parental species play important role in broadening and improving the genetic basis of *B*. *napus* by the introgression of genetic resources from parental species. With these strategies, it is easy to select new types of *B*. *napus*, but difficult to select new types of *B*. *rapa* or *B*. *oleracea* by self-pollination. This characteristic may be a consequence of high competition with *B*. *napus* gametes. To verify the role of gamete viability in producing new *B*. *napus* individuals, the meiotic chromosome behavior of the interspecific hybrid between *B*. *napus* (Zhongshuang 9) and *B*. *oleracea* (6m08) was studied, and microspore-derived (MD) individuals were analyzed. The highest fitness of the 9:19 (1.10%) pattern was observed with a 5.49-fold higher than theoretical expectation among the six chromosome segregation patterns in the hybrid. A total of 43 MD lines with more than 14 chromosomes were developed from the hybrid, and 8 (18.6%) of them were *B*. *napus*-like (n = 19) type gametes, having the potential to broaden the genetic basis of natural *B*. *napus* (GD = 0.43 ± 0.04). It is easy to produce *B*. *napus*-like gametes with 19 chromosomes, and these gametes showed high fitness and competition in the microspore-derived lines, suggesting it might be easy to select new types of *B*. *napus* from the interspecific hybrid between *B*. *napus* and *B*. *oleracea*.

## Introduction

*Brassica oleracea* is an important vegetable crop and is genetically diverse, having various subspecies, such as cabbage, cauliflower, broccoli, kale and wild-type, and having many known useful traits, such as its strong resistance against *Sclerotinia* incorporated from wild subspecies of *B*. *incana* [1, 2]. *B*. *napus* is an important oilseed crop in the world, originating from a natural interspecific hybridization between *B*. *rapa* and *B*. *oleracea* ~6000 years ago [3, 4]. This crop’s genetic basis was narrower than the parental species due to its short history and domestication through modern breeding methods [5]. Introgression of genetic resources from parental species into *B*. *napus* is necessary to broaden and improve its genetic basis [6–9].

To utilize the genetic resources of parental species, the strategy of crossing *B*. *napus* and its parental species is commonly used. In the strategy, it is easy to select new types of *B*. *napus*, either gaining useful traits from parental species [10, 11] or having the potential to broaden the genetic basis of natural *B*. *napus* [8, 12, 13]. However, it is difficult to select new types of *B*. *oleracea/B*. *rapa* individuals, which might due to higher competition of *B*. *napus* gametes than *B*. *oleracea/B*. *rapa* gametes. To verify this hypothesis, the meiotic behavior of interspecific hybrid between *B*. *napus* and *B*. *oleracea* and its microspore-derived (MD) individuals were analyzed.

Microspore culture is widely applied in *Brassica* species to produce double haploid (DH) individuals in germplasm collection, QTL mapping, genetic engineering and crop improvement [14–17]. This method is less commonly used in interspecific hybrids between *Brassica* species due to the difficulty in obtaining embryoids [18–20]. However, scientists have used the technique in interspecific hybrids to induce microspore-derived lines, aiming to study male meiotic behavior, since there is no selection pressure from females compared with self-pollination and backcrossing [21, 22]. In the present study, an interspecific hybrid between *B*. *napus* and *B*. *oleracea* was developed, and its meiotic behavior and gamete behavior in microspore-derived individuals were analyzed, showing that the *B*. *napus*-like gamete had high fitness and competition in the hybrid. This suggested that high viability of *B*. *napus*-like gametes might make it easy to select new types of *B*. *napus* from the interspecific hybrid between *B*. *napus* and its parental species by self-pollination.

## Materials and methods

### Plant materials

The interspecific hybrid ACC was developed from hybridization between *B*. *napus* ‘Zhongshuang 9’ and *B*. *oleracea* ‘6m08’ via embryo rescue and propagated on MS regeneration medium (MS + 3 mg/L 6-BA + 0.02 mg/L NAA) via tissue culture for microspore culture [23]. Morphology, fertility, chromosome number and genetic components of MD lines were evaluated, and their genetic diversity was compared with 34 natural *B*. *napus* and 42 *B*. *oleracea* (S1 Table).

### Cytological observations

#### Chromosome number at mitotic metaphase

To check the chromosome numbers of the ACC hybrid and MD progenies, the young ovaries were collected and pretreated with 2 mmol/L 8-hydroxyquinoline for three to four hours at room temperature and later fixed in Carnoy’s solution (V_ethanol_: V_acetic acid_ = 3:1) and stored at 4 °C. Mitotic observations were made according to the methods as described by Li et al. [24]. The ovaries at mitosis were hydrolyzed in 1 M HCl at 60 °C for 8 min and stained with 10% modified carbol fuchsin and observed under microscope.

#### Chromosome pairing and segregation at meiosis

For meiotic analysis, buds were fixed in Carnoy’s solution for 24 h and then transferred into fresh mixture and stored at –20 °C for future use. Meiotic observations of pollen mother cells (PMCs) were made according to the methods of Li et al. [24]. The anthers at meiosis stage were hydrolyzed in 1 M HCl at 60 °C for 2 min, stained with 10% modified carbol fuchsin and observed under microscope. The chromosome pairing at metaphase I and chromosome segregation at anaphase I in PMCs were recorded.

### Pollen fertility

Pollen fertility was determined by the percentage of pollen grains stained with 1% acetocarmine according to the method of Li [24]. Three flowers were counted from ACC hybrid and MD lines. More than 300 pollen grains were recorded for each line. Grains that were round and stained red were considered normal, whereas small and non-stained ones were considered dead pollen.

### Microspore isolation

Microspore culture was performed by the method described by Lichter [25], with minor modifications. A total of 30 flower buds ranging in length from 2.5 to 3.5 mm from the ACC hybrid were selected and sterilized in 10% sodium hypochlorite solution for 15 min. The sterilized buds were then released with B5-13 medium. The solution along with the microspores were filtered through a 48-μm filter and transferred into a sterile 10 mL centrifuge tube, and the volume was adjusted to 8 mL with B5-13 media. The microspores were then centrifuged for 3 min at 1200 rpm, and the supernatant was discarded. B5-13 media was added to mix the microspores, and then they were centrifuged for 3 min at 1200 rpm again. The supernatant was discarded and microspores were re-suspended in 8 mL NLN-13 solution (NLN medium plus 13% sucrose in Millipore water, pH to 5.8).

The microspore suspension was divided into 4 Petri dishes with diameter of 70 mm, and 4 mL NLN-13 and 1 mL 10% activated charcoal were added into each Petri dish, which were later sealed with parafilm. The isolated microspores were incubated at 32 °C for 48 hours and then transferred into a 24 °C incubator for 20 days [26]. The plates were then put on a shaker (60 rpm) for embryo development. Three weeks later, young embryos were transferred into ½ MS medium for plant induction.

### SSR marker analysis

Genomic DNA was isolated from young leaves using the CTAB method [27]. 30 MD lines randomly selected were genotyped with 34 natural *B*. *napus* and 42 *B*. *oleracea* using 35 sets of SSR primers (S2 Table). The SSR results were described by the absence (0) or presence (1) of a band.

The genetic distance (GD) between accessions *X* and *Y* was calculated using the formula, GD*xy* = 1 –N*xy* / (N*x* + N*y*), where N*xy* is the number of common bands shared by accession *X* and *Y*, and N*x* and N*y* are the total number of bands in accession *X* and *Y*, respectively [28]. The phylogenetic tree was constructed using the neighbor-joining method implemented by MEGA version 6 [29].

### Statistical analysis

Analysis of variance (ANOVA), Pearson’s simple correlation coefficient and *X*^*2*^ test were calculated using the statistical package SAS version 8.0 [30].

## Results

### Development of interspecific hybrid between *B*. *napus* and *B*. *oleracea*

**Fig 1:**
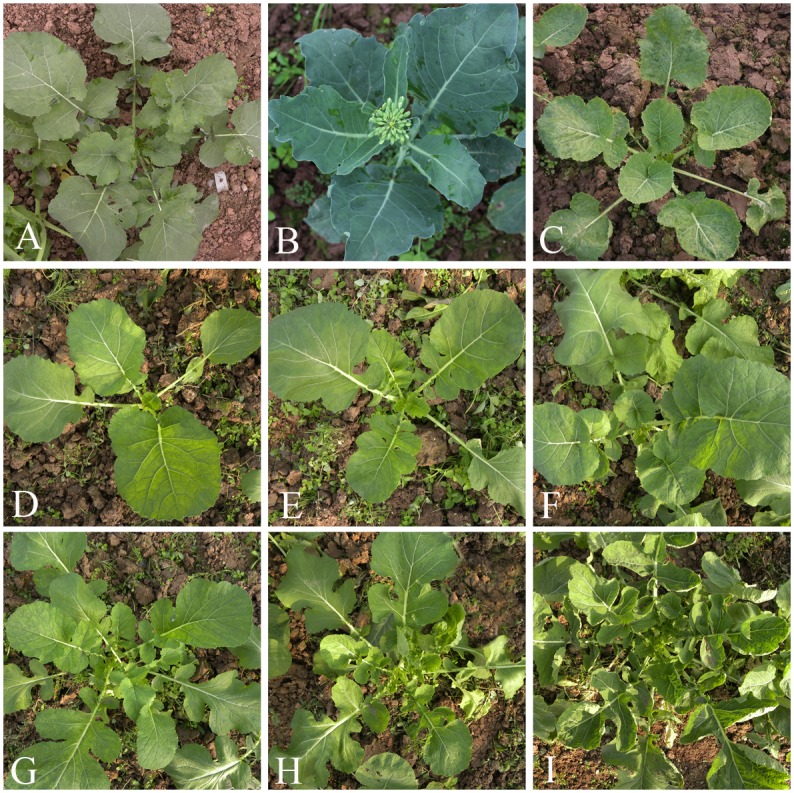
Morphology of the ACC interspecific hybrid between *B*. *napus* and *B*. *oleracea* and its microspore-derived lines. (A) Zhongshuang 9 seedling; (B) 6m06 seedling; (C) hybrid ACC seedling; (D-I) Seedling of microspore-derived lines from the hybrid between Zhongshuang 9 and 6m08.

**Fig 2:**
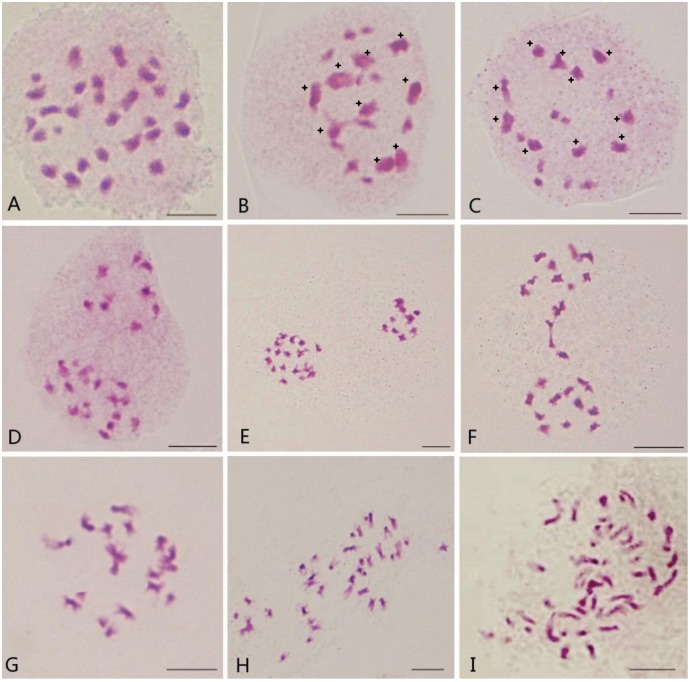
Cytology of the ACC hybrid and its microspore-derived lines from *B*. *napus* and *B*. *oleracea*. (A) One cell of ACC with chromosome number 28; (B) One PMC of ACC with 9II + 10I; (C) One PMC of ACC with 10II + 8I; (D) One PMC of ACC with 9:19; (E) One PMC of ACC with 10:18; (F) One PMC of ACC with chromosome bridge; (G) One microspore-derived line with 18 chromosomes; (H) One microspore-derived line with 38 chromosomes; (I) One microspore-derived line with 52 chromosomes. Those marked with stars were bivalent.

Immature embryos 7~10 days after pollinating with *B*. *oleracea* (6m08) pollen on the stigma of *B*. *napus* (Zhongshuang 9) were cultured on ½ MS medium via embryo rescue. Three weeks later, an interspecific hybrid was developed, sharing intermediate morphology between two parents and having lighter green leaf color than both parents (Fig 1A–1C). Its pollen fertility was 34.82%, which was lower than the parental species (Zhongshuang 9: 99.5%, 6m08: 96.4%), and its chromosome number was 28 in meiotic and mitotic cells (Fig 2A).

### Meiotic behavior of an interspecific hybrid between *B*. *napus* and *B*. *oleracea*

Different chromosome conformations, such as univalents, bivalents, trivalents and quatrivalents, were observed in pollen mother cells (PMCs) at metaphase I (MI) of the hybrid. The average chromosome association was 9.66I + 9.12II + 0.01III + 0.02IV in 170 PMCs at MI. In certain cases, the frequency of the pattern of 9II + 10I (84.71%) was higher than the pattern of 10II + 8I (12.84%) (Fig 2B and 2C). Despite the high frequency of chromosome segregations of 13:15 (36.81%), 12:16 (30.77%) and 14:14 (15.93%) patterns were observed in 182 PMCs at anaphase I (AI), the fitness of the 9:19 (1.10%) pattern was the highest among the 6 patterns observed with a 5.49-fold higher fitness than the theoretical expectation (Fig 2D and 2E). This finding suggested that there would be a high probability of producing *B*. *oleracea*/*B*. *napus*-like gametes (C/AC = 9:19). In certain cases, meiotic irregularities, such as chromosome bridges and lagging chromosomes, were observed during the first and second divisions in the hybrid (Fig 2F).

### Development of microspore derived plants

In total, 115 embryoids (18.55%) were induced from 620 flower buds between late uninucleate stage and early binucleate stage (2.5~3.5mm flower bud) of the hybrid via microspore culture. Only 43 MD lines were obtained after transplanting these embryoids on ½ MS medium for plant-induction. All of these lines shared light green leaf color with the hybrid but had different number of leaf auricles, for example, the number of the leaf auricles ranged from 0 to 5 (Fig 1D–1I).

### Pollen fertility and chromosome number of microspore-derived plants

Pollen fertility of the MD lines ranged from 0 to 98.89%, with an average of 49.42%. Fertility was significantly positively correlated with chromosome number (*P* = 0.0027, *r* = 0.70; S3 Table), suggesting that lines with more chromosomes had higher pollen fertility.

In the 43 MD individuals, diverse chromosome numbers were observed. With the exception of one individual having 66 chromosomes and one having more than 80 chromosomes, the chromosome number of the other 41 individuals ranged from 15 to 56. Of these individuals, 14 were haploid, and 29 were polyploid by natural chromosome doubling. In detail, five of them had 17 chromosomes, five had 38 chromosomes, four had 30 chromosomes, four had 56 chromosomes and three lines had 19 chromosomes. Overall, twelve patterns of gametes were found. The frequency of actual gametes was significantly different from the theoretical gametes via *X*^*2*^ test (*P* < 0.0001). This analysis showed that all the individuals had more than 14 chromosomes, suggesting that gametes having more chromosomes might survive, whereas the ones with less might die during the meiosis stage in the interspecific hybrid between *B*. *napus* and *B*. *oleracea*.

Although 27 (65.85%) individuals were aneuploid (n ≠ 19), five (12.20%) individuals were unreduced gametes (n = 28), individuals having gametes with 19 chromosomes (19.51%, 8/41) were the most common of all the patterns (Fig 2G–2I). This indicated that *B*. *napus*-like individuals having gametes with 19 chromsomes were more competitive than others in the hybrid between *B*. *napus* and *B*. *oleracea*.

### Genetic diversity of microspore derived plants

To verify the genetic diversity of the MD population, 115 polymorphic loci were amplified by genotyping 30 MD individuals with 35 combinations of SSR primers. Compared to the parental species (Zhongshuang 9 and 6m08), the MD population shared on average ~53 loci (45.71 ± 1.11%) with both parental species, ~37 loci (32.26 ± 1.60%) with the single parent *B*. *napus* (Zhongshuang 9) and ~14 (12.09 ± 1.60%) with the single parent *B*. *oleracea* (6m08). However, these plants also had ~11 unique loci (9.94 ± 1.11%) distinct from both parents. The average genetic distance between the MD population and the *B*. *oleracea* parent (0.91 ± 0.07) was significantly farther than the *B*. *napus* parent (0.34 ± 0.05, *P* < 0.0001). Compared to Zhongshuang 9, the genetic distance of *B*. *napus*-like individuals (0.39 ± 0.07, *P* = 0.046) was significantly more distant than aneuploid (0.33 ± 0.04) and unreduced gametes (0.32 ± 0.02). This finding was similar to the distance between MD lines and 6m08 (*B*. *napus*-like individuals: 0.94 ± 0.09; aneuploid: 0.91 ± 0.07; unreduced gamete: 0.88 ± 0.05; Table 1), suggesting more genetic components from the *B*. *napus* parent than the *B*. *oleracea* parent were inherited by the MD individuals.

**Table 1:** Comparing the genetic distance of *B*. *napus*-like individuals, aneuploid and unreduced gametes of microspore-derived lines to the parental species, natural *B*. *napus* and *B*. *oleracea*.

	*B*. *napus*-like*	Aneuploid	Unreduced gamete
Zhongshuang 9	0.39 ± 0.07	0.33 ± 0.04	0.32 ± 0.02
6m08	0.94 ± 0.09	0.91 ± 0.07	0.88 ± 0.05
AACC group	0.43 ± 0.04	0.39 ± 0.03	0.38 ± 0.02
CC group	0.94 ± 0.05	0.94 ± 0.04	0.93 ± 0.04

*: *B*. *napus*-like individuals with n = 19 chromosomes.

**Fig 3:**
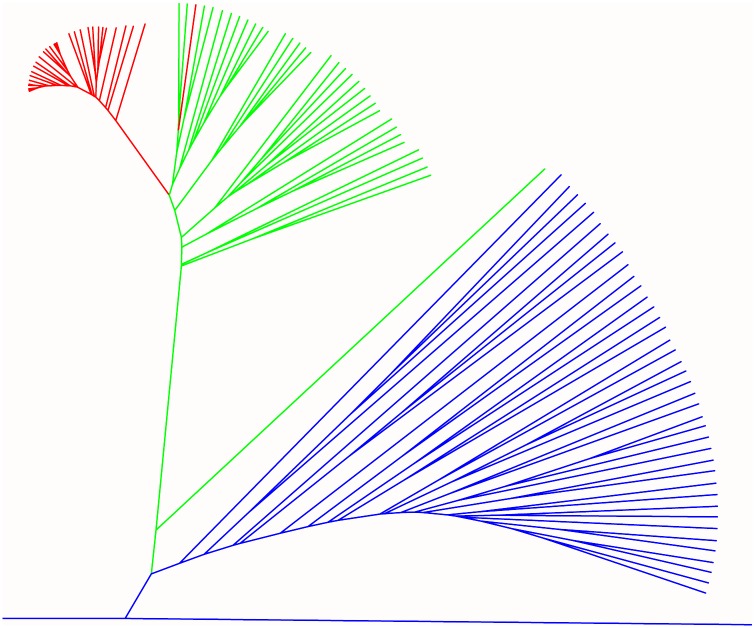
Phylogenetic trees showing the relationships between 30 MD progeny (red lines) and 42 *B*. *oleracea* (blue lines), 34 *B*. *napus* (green lines).

This finding was in accordance with the distance among MD population, natural *B*. *napus* and *B*. *oleracea*. In comparison with 34 *B*. *napus* and 42 *B*. *oleracea* subspecies, the average genetic distance between MD population and *B*. *oleracea* population (0.97 ± 0.37) was similar to that between *B*. *napus* and *B*. *oleracea* population (0.97 ± 0.33), but it was further than that between MD population and *B*. *napus* population (0.42 ± 0.17), suggesting the MD population is different from natural *B*. *napus* and *B*. *oleracea*, but close to *B*. *napus*. The obvious genetic differences among MD lines, *B*. *napus* population and *B*. *oleracea* population were also supported by the phylogenetic tree (Fig 3). Although the average genetic distance among *B*. *napus*-like individuals, aneuploid and unreduced gametes were similar, the genetic distance of *B*. *napus*-like individuals to the natural *B*. *napus* group (0.43 ± 0.04, *P* = 0.0091) was farther than that of aneuploid (0.39 ± 0.03) and unreduced gametes (0.38 ± 0.02; Table 1). This finding indicated that these *B*. *napus*-like individuals, having gametes with 19 chromosomes, had the potential to widen the genetic basis of *B*. *napus*.

## Discussion

### Meiotic behavior of *Brassica* interspecific hybrid revealed by microspore culture

Interspecific hybridization plays an important role in exchanging genetic components, widening and improving genetic resources in *Brassica* species. Although high frequency of euploids (new type *B*. *napus*) was observed in the interspecific hybrid between *B*. *napus* and parental species [8, 9, 20, 31, 32], aneuploid and unreduced gametes occurred frequently due to abnormal meiosis of interspecific hybrids [33–36]. In the present study, only 43 individuals were developed from the interspecific hybrid between *B*. *napus* and *B*. *oleracea* due to the difficulty in generating a large number of microspore-derived lines, and these individuals exhibited 19.51% euploid, 65.85% aneuploid and 12.20% unreduced gametes.

The frequency of aneuploid, euploid and unreduced gametes in the interspecific hybrid might be attributable to genotype-specific effects, such as sharing a common subgenome, or environmental factors, such as cold or fluctuating temperatures, plant nutrition, water stress and disease [37–42]. In the present study, the interspecific hybrid sharing a common C-subgenome from *B*. *napus* and *B*. *oleracea*, and produced high frequency of euploid (19.51% *B*. *napus*-like gametes), which was similar to the interspecific hybrid between *B*. *napus* and *B*. *rapa* sharing an A-subgenome [20]. It is necessary to investigate the genetic or developmental factors that may give rise to this apparent selection for the variation of gametes in the interspecific hybrid between *B*. *napus* and *B*. *oleracea* in the future.

### Genetic variance of microspore derived lines

In interspecific hybridization, chromosomes of related species recombine and interact regularly, causing homoeolog expression bias, genomic dominance and genomic imprinting [43–45]. In the MD lines, the genetic distance was closer to the *B*. *napus* parent (0.34 ± 0.05) than the *B*. *oleracea* parent (0.91 ± 0.07), suggesting more genetic components of *B*. *napus* than *B*. *oleracea* were inherited into the MD population. This might be due to genomic dominance and genomic imprinting of the *B*. *napus* parent in the MD lines. Subgenome dominance is an important phenomenon in allopolyploids, it was also observed in the interspecific hybrids. For example, in the interspecific hybrids (wheat × *Aegilops*), C-subgenome nucleolar organizing regions loci are dominant [46]. In addition, the subgenome dominance occurred instantly following the hybridization [47]. This bias in gene expression must be investigated in exploring the mechanism of *B*. *napus* genomic dominance.

In the present study, all of the MD lines were different from the parental species, especially the *B*. *napus*-like individuals, which has the potential to broaden the genetic basis of natural *B*. *napus*. The other lines might be used to produce monosomic alien addition lines and nullisomic lines, which can be used as bridge to transfer desired genes from wild *B*. *oleracea* species into *B*. *napus* [48, 49]. The role of these novel MD plants in *Brassica* species improvement needs to be evaluated in the future.

## Supporting information

S1 Table Accessions of 34 natural *B*. *napus* and 42 *B*. *oleracea* used to analyze genetic diversity of microspore-derived lines derived from the hybrid between *B*. *napus* and *B*. *oleracea*. (XLSX)

S2 Table List of SSR primers. (XLSX)

S3 Table Data from microspore-derived lines derived from interspecific hybrid between *B*. *napus* and *B*. *oleracea* relating to fertility, chromosome number and genetic variance. (XLSX)
